# Short-Wavelength Light Enhances Cortisol Awakening Response in Sleep-Restricted Adolescents

**DOI:** 10.1155/2012/301935

**Published:** 2012-07-30

**Authors:** Mariana G. Figueiro, Mark S. Rea

**Affiliations:** Lighting Research Center, Rensselaer Polytechnic Institute, 21 Union Street, Troy, NY 12180, USA

## Abstract

Levels of cortisol, a hormone produced by the adrenal gland, follow a daily, 24-hour rhythm with concentrations reaching a minimum in the evening and a peak near rising time. In addition, cortisol levels exhibit a sharp peak in concentration within the first hour after waking; this is known as the cortisol awakening response (CAR). The present study is a secondary analysis of a larger study investigating the impact of short-wavelength (*λ*_max_ ≈ 470 nm) light on CAR in adolescents who were sleep restricted. The study ran over the course of three overnight sessions, at least one week apart. The experimental sessions differed in terms of the light exposure scenarios experienced during the evening prior to sleeping in the laboratory and during the morning after waking from a 4.5-hour sleep opportunity. Eighteen adolescents aged 12–17 years were exposed to dim light or to 40 lux (0.401 W/m^2^) of 470-nm peaking light for 80 minutes after awakening. Saliva samples were collected every 20 minutes to assess CAR. Exposure to short-wavelength light in the morning significantly enhanced CAR compared to dim light. Morning exposure to short-wavelength light may be a simple, yet practical way to better prepare adolescents for an active day.

## 1. Introduction

Circadian rhythms in mammals are endogenously driven physiologic oscillations that are generated by a master neural clock located in the suprachiasmatic nuclei (SCN) of the hypothalamus [1]. The SCN respond to retinal light exposure and can be entrained to the local solar day by regular exposures to a 24-hour rhythm of light and dark. It is now well accepted that the circadian system is maximally sensitive to short-wavelength (blue) light (peak sensitivity close to 460 nm) as measured by acute melatonin suppression or phase shifting of the dim light melatonin onset (DLMO) [2–4]. Short-wavelength light has also been shown to increase heart rate and alertness at night [5, 6] and to induce an increase in clock gene PER 2 expression in the evening [7]. The impact of short-wavelength light on cortisol secretion is more poorly understood.

Levels of cortisol, a hormone synthesized by the cortex of the adrenal gland, follow a daily, 24-hour rhythm [8, 9]. Its concentration is low throughout the day, reaching a broad minimum in the evening before rising slowly again throughout the night. In addition to this gradual elevation of cortisol throughout the night-time, cortisol levels sharply increase from 30 to 60 minutes after awakening; this increase is known as the cortisol awakening response (CAR) [10–14]. Conversely, the spike in corticosteroids in nocturnal rodents is associated with the start of activity at night [15]. In general, the highest corticosteroid concentrations in blood and saliva are associated with the time of transition from rest to activity (i.e., waking) in both nocturnal and diurnal species [15, 16]. A high CAR has been hypothesized to reflect the anticipation of stress [17]; therefore, a reduced cortisol secreted after awakening may result in decreased ability to deal with environmental stressors. 

The impact of sleep restriction on CAR has also been reported. One study showed that CAR is diminished in 2- to 4-year-old children after sleep restriction [18]. It has also been demonstrated, under laboratory conditions, that postawakening cortisol levels are reduced when young adults are sleep deprived for 26 hours compared to sleeping for 7 hours [19]. Consistently, Späth-Schwalbe et al. reported higher releases of cortisol after long sleep durations as compared to short sleep, which was measured using polysomnography (PSG) [20]. Griefahn and Robens confirmed these results by showing that total sleep time, as measured by PSG, was positively correlated with CAR in evening types after sleep during the nighttime but not after sleep during the daytime [21]. 

Morning exposure to polychromatic (white) light has been shown to enhance CAR in humans who were not sleep restricted. Leproult et al. showed that morning exposure (05:00–08:00 h) to a bright light (5,000 lux of a white light at participants' corneas) resulted in a 50% increase in cortisol levels compared to remaining in dim light (<150 lux). Afternoon exposure (13:00–16:00 h) to the same light did not impact cortisol levels [22]. Scheer and Buijis also showed an effect of morning light exposure, but not evening light exposure, on cortisol levels and on heart rate. A 1-hour exposure to 800 lux of white light in the morning resulted in a 35% increase in cortisol levels compared to dim light. Evening cortisol was not affected by light exposure [23]. More recently, Jung et al. showed an acute suppressive effect on cortisol levels following bright light exposure (10,000 lux of white light for 6.5 hours) during the biological night and morning, close to the subjects' habitual wakeup times [24]. 

The present paper represents a subanalysis of a larger unpublished study investigating the impact of evening and morning short-wavelength light on performance and on biomarkers in sleep-restricted adolescents. Here, we report the impact 40 lux (0.401 W/m^2^) of a 470-nm peaking (blue) light on CAR in sleep-restricted adolescents, aged 12–17 years. We hypothesized that the 470-nm light would significantly enhance CAR in sleep-restricted adolescents compared to dim light exposure.

In real-life situations, adolescents can be chronically sleep deprived because of their inability to fall asleep early and their fixed wakeup times during school days. Based on the literature, it is reasonable to hypothesize that CAR in adolescents may be diminished as a result of the reduced total sleep time; therefore, an enhancement in CAR by morning light has the potential to better prepare sleep-restricted adolescents for daily environmental stressors.

## 2. Methods

### 2.1. Participants

Eighteen adolescents, nine females, mean ± standard deviation (SD) age 13.4 ± 1.0 years, and nine males, average ± SD age 16.5 ± 0.7 years, were recruited to participate in the study (mean age ± SD 15 ± 1.8 years). All subjects signed an assent form and parents signed a consent form approved by the Rensselaer Polytechnic Institute Institutional Review Board and were paid for their participation. Participants were divided into two groups based on gender, and each group of nine participants came to the laboratory for three overnight sessions spaced one week apart. All participants completed a Munich Chronotype Questionnaire (MCTQ) [25]. Mean ± SD chronotypes of the female and male participants were 3.2  ±  0.4 and 2.7  ±  1.7, respectively (overall mean ± SD chronotype was 2.9 ± 1.2). Chronotypes for each participant are shown in Table 1. Participants were asked to keep a regular sleep/wake schedule during the three weeks of the study (bedtimes no later than 23:00 h and wake times no later than 07:00 h) and were asked to keep a daily sleep log. None of the participants reported having sleep problems. All participants wore a Dimesimeter mounted on a wrist band to measure light exposures and to verify the regularity of their activity-rest periods during the three weeks of the study [26]. The Dimesimeter is a small, unobtrusive, and user friendly research tool for collecting personal light exposures and activity levels over multiple days and nights. The Dimesimeter is calibrated in terms of the spectral sensitivities of the visual and circadian systems [26]. Based on the sleep logs and on the Dimesimeter data, all participants complied with the sleep schedules.

### 2.2. Lighting Conditions

Special long-wavelength (red) and short-wavelength (blue) light goggles were constructed for the study. Four long-wavelength (*λ*_max⁡_ ≈ 630 nm) or four short-wavelength (*λ*_max⁡_ ≈ 470 nm), light emitting diodes (LEDs) were mounted on the lenses of transparent goggles. The long-wavelength, red light goggles were used in the evening, as detailed below. For both spectra, one LED was mounted above and one was mounted below the center each of the two lenses. To limit the luminance of the sources, and thus minimize the risk of blue light hazard [27], a small diffuser was placed in front of each LED. The light goggles used a 9-V battery, which was removed from a controller box when the goggles were not being used.

Light goggles were calibrated prior to each experimental session using a spectrometer (Oriel Multispec model 77400 with an Oriel Instaspec IV CCD detector). An opal diffuser was fixed over the fiber-optic input to the spectrometer to produce the needed spatial response for measuring irradiance. The spectrometer was first calibrated for wavelength accuracy using four visible spectrum mercury emission lines from a cool-white fluorescent lamp (GE F15T8-CW) and the 632.8 nm emission line from a helium-neon laser (Melles Griot, model 05-LHP-141). The output of the spectrometer was calibrated for spectral irradiance (W/(m^2^·nm)) from readings taken at a prescribed distance (1.00 m) from a tungsten-halogen lamp standard (lamp no. 12, 75 W Q/CL) traceable to the National Institute of Standards and Technology (NIST). Each pair of goggles was then placed in a measurement jig that held it approximately 2 cm (the typical distance between a participant's cornea and the goggle lenses) from the spectrometer input diffuser. Spectral irradiance levels were iteratively measured to reach 40 lux (0.401 W/m^2^ for 470 nm and 0.182 W/m^2^ for 630 nm). This same procedure was used for both the short- and long-wavelength goggles. Spectral irradiances were equated for photopic illuminance because there is no known model for the spectral sensitivity of the adrenal gland to light. Our starting point for research was to equate the short- and the long-wavelength lights for equal “visual” effectiveness.

### 2.3. Procedures

The study was run over the course of three overnight sessions, at least 1 week apart. The three experimental sessions differed in terms of the light exposure scenarios experienced during the evening prior to sleeping in the laboratory and during the morning after awaking.  Table 2 shows the three lighting scenarios that were presented to all 18 participants. Light Sessions 1 and 2 differed only in their evening light exposures (exposure to either dim or long-wavelength light), but not in their morning light exposures (always short-wavelength light exposure). Long-wavelength light exposure in the evening was used because it does not phase shift the circadian clock, but it may increase alertness, and therefore, improve performance at night [28]. 

Participants arrived at the laboratory at 22:30 h after eating a normal meal at home. The participants in Light Session 1 wore the long-wavelength light goggles starting at 22:45 h and continued wearing them until the end of all testing at 01:15 h. All participants were asked to perform two 60-minute performance tests twice, starting at 23:00 h and 00:15 h. One performance test was measured on the Multi-Attribute Task Battery (MAT) for Human Operator Workload and Strategic Behavior Research software program (NASA COSMIC collection, Open Channel Foundation). The 54-minute MAT Battery was comprised of (i) a monitoring task, (ii) a tracking task, and (iii) a resource management task. The second set of tests included short-term memory and reaction times tests that took about 6 minutes to complete. Although our short-term performance tests showed a slight improvement with morning short-wavelength light, none of the differences reached statistical significance, and those results will not be further discussed here.

At the end of the evening sessions (01:15 h), participants were given a prescribed, fixed meal. It is important to note that, although a meal in the middle of the night may be a zeitgeber, the same procedure was repeated in all three weeks of the experiment; therefore, this variable was constant throughout the experiment. Participants then retired to a sleeping area in the laboratory at 01:30 h and were awakened at 06:00 h Sunday morning; the opportunity to sleep in a dark room was 4.5 hours. Upon awakening at 06:00 h, saliva samples were collected in dim light, while participants were still on their mattresses. Soon after the first sample collection, they were asked to remain awake in bed and were presented either the short-wavelength light or the dim light. Participants were allowed to engage in conversations and to quietly listen to music. In every experimental week, 6 participants were presented with the short-wavelength light while 3 participants remained in dim light. Saliva samples were obtained every 20 minutes from waking at 06:00 h until 07:20 h, at which time they had a 10-minute break. A total of 5 samples were collected while participants remained on their mattresses. After the break, participants began the 60-minute performance tests at 07:30 h. Two additional samples were collected just before and just after they performed the tests, and these samples were not included in the analyses because the tests themselves could have influenced cortisol levels. The experiment ended at 08:30 h.

### 2.4. Sample Collection and Data Analyses

Saliva samples were collected for subsequent concentration level assessments of cortisol using the Salivette system from Alpco Diagnostics (Salem, NH, USA). This system consists of a centrifuge vessel with a suspended insert in which a cotton swab is placed. To collect the saliva, the cap was removed and the participants put the tube against the lips to take the cotton swab into the mouth without touching it with the fingers. The participants then chewed the swab to impregnate it with saliva. Between 1-2 mL of saliva are required for the analyses. After chewing the cotton, the participants then spit the cotton back into the suspended insert, and the cap on the tube was replaced. The vessels containing the suspended saliva-impregnated cotton swabs were then spun in a centrifuge at 3500 rotations per minute (1000 ×g) for five minutes, causing the saliva to collect at the bottom of the centrifuge vessel. Saliva samples were then frozen for transport to a laboratory for cortisol assays (Pharmasan, Osceola, WI, USA). The limit of detection for the cortisol assay was 0.0036 *μ*g/dL and the intra- and interassay coefficients of variability were 3.6% and 6.4%, respectively.

To ascertain consistency in results, three analyses were performed using the cortisol levels data. The first one was used to examine the impact of light on CAR. For this analysis, CAR was calculated as the difference between the cortisol level at 20 and at 40 minutes relative to the time of awaking. Two-tailed, paired Student's *t*-tests were used to determine whether CAR under Light Sessions 1 and 2 were significantly different from those obtained during the Dim Light Session.

For the second analysis, the area under the curve (AUC) with respect to the increase in cortisol (i.e., with reference to the postawakening, first sample collected in dim light) was calculated based on the method described by Pruessner et al. [29]. We used the unnormalized data and included in the calculations the postawakening samples (always collected in dim light) and the samples collected at 20, 40, 60, and 80 minutes postawakening (collected under one of the three lighting conditions). Two-tailed, paired Student's *t*-tests were used to compare the AUC values in the Dim Light Session to those in Light Sessions 1 and 2.

For the third analysis, cortisol levels for each participant were normalized to their 06:00 h data point (collected in dim light) for each session. This normalization was performed to account for any week-to-week variability in the postawakening cortisol levels. Based on Clow et al. [12], we had no reason to believe that the first sample collected in dim light upon awakening would be different between weeks, and individual differences were not of interest here.

A three (lighting sessions) by four (sample times) repeated measures analysis of variance (ANOVA) was performed on the normalized data using samples collected at 20, 40, 60, and 80 minutes postawakening. Two-tailed, paired Student's *t*-tests were performed to examine the main effects and interactions. When applicable, a Bonferroni correction was applied to the multiple *t*-test comparisons. All statistical analyses were performed using PASW Statistics 18.0.

## 3. Results

Results from the first analysis showed that CARs at 20 minutes were significantly greater in Light Sessions 1 and 2 than in the Dim Light Session (*t*(17) = 2.3, *P* = 0.04 and *t*(17) = 2.8, *P* = 0.01, resp.). The mean ± standard error of the mean (SEM) CAR (defined as the difference in cortisol levels at waking and at 20 minutes post-awakening) were 0.14 ± 0.03 *μ*g/dL (3.9 ± 0.8 nmol/L) for the Dim Light Session, 0.23 ± 0.04 *μ*g/dL (6.4 ± 1.2 nmol/L) for Light Session 1, and 0.24 ± 0.03 *μ*g/dL (6.5 ± 0.9 nmol/L) for Light Session 2. CARs at 40 minutes after awakening were not significantly greater in Light Sessions 1 and 2 compared to CARs at 40 minutes in the Dim Light Session (*t*(17) = 1.4, *P* = 0.17 and *t*(17) = 1.9, *P* = 0.07, resp.). Mean ± SEM CARs at 40 minutes after awakening (defined as the difference in cortisol levels at waking and at 40 minutes post-awakening) were 0.15 ± 0.06 *μ*g/dL (4.2 ± 1.6 nmol/L) for the Dim Light Session, 0.24  ±  0.06 *μ*g/dL (6.6 ± 1.6 nmol/L) for Light Session 1, and 0.29 ± 0.06 *μ*g/dL (7.9 ± 1.8 nmol/L) for Light Session 2.

**Figure 1:**
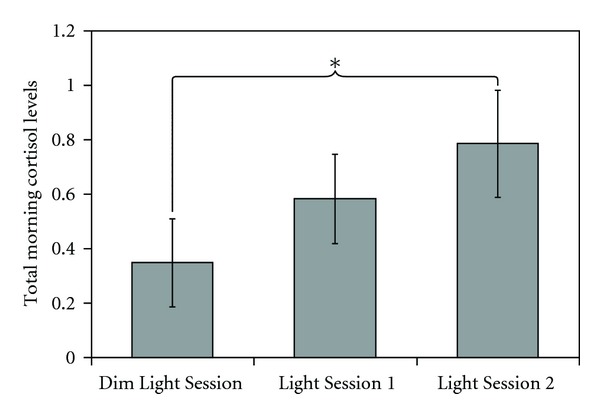
Total morning cortisol levels during the 80 minutes of data collection [(area under the curve (AUC)] were lower during dim light than during blue light exposure. Although the AUC was greater during blue light exposures than during the Dim Light Session, the difference was statistically significant only for the second session. Shown are the mean ± SEM total cortisol levels (AUC for 80 minutes) for each experimental condition.

Figure 1 shows the mean ± SEM total cortisol levels during the 80-minute data collection period (AUC) for each experimental condition. The two-tailed, paired Student's *t*-tests revealed a significantly greater AUC for Light Session 2 than for the Dim Light Session (*t*(17) = 2.3, *P* = 0.04). The AUC for Light Session 1 was greater, but not significantly different, than the AUC for the Dim Light Session (*t*(17) = 1.5, *P* = 0.16). The mean ± SEM AUC was 0.35 ± 0.2 *μ*g/dL (9.6  ±  5.5 nmol/L) for the Dim Light Session, 0.58  ±  0.2 *μ*g/dL for Light Session 1 (16  ±  5.5 nmol/L), and 0.78  ±  0.8 *μ*g/dL for Light Session 2 (21.5 ± 5.5 nmol/L).

**Figure 2:**
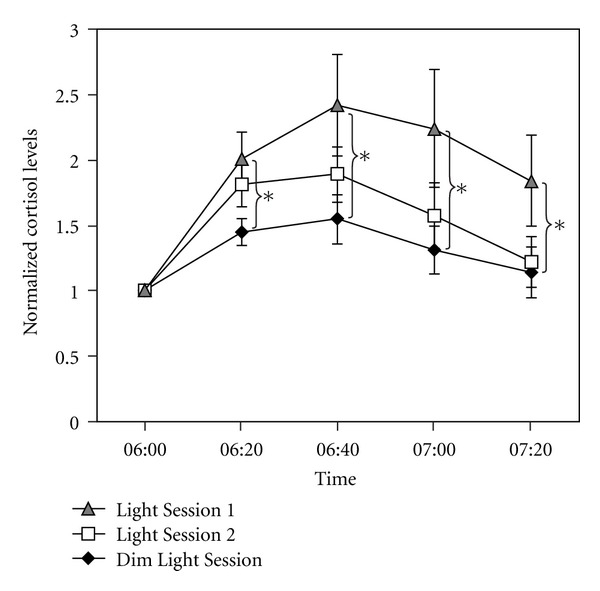
Normalized morning cortisol levels during the Dim Light Session and during the two blue light exposure sessions. All data were normalized to levels gathered at the first, common experimental sampling time of 6:00 AM, which was the time of awakening. Shown are the mean ± SEM normalized cortisol levels for each experimental condition and sample collection time; normalized cortisol levels during blue light exposures that were significantly different than those during dim light are indicated with asterisks.

Figure 2 shows the normalized mean ± SEM cortisol levels for each lighting scenario at each sample collection time. The ANOVA revealed a significant main effect of lighting session (*F*_2,34_ = 4.5; *P* = 0.02), a significant main effect of time (*F*_3,51_ = 8.7, *P* < 0.0001), and a significant interaction between the variables (*F*_6,102_ = 2.4, *P* = 0.03). As with the AUC, the two-tailed, paired Student's *t*-tests revealed that, compared to the Dim Light Session, there was a significant increase in cortisol levels in response to light during Light Session 2 (*t*(17) = 2.5, *P* = 0.02), but not during Light Session 1 (*t*(17) = 1.5, *P* = 0.1). Based upon the significant interaction, the *post hoc*, two-tailed, paired Student's *t*-tests also revealed that cortisol levels for the Dim Light Session were significantly lower than those under Light Session 2 at 20 minutes after awakening (*t*(17) = 2.7, *P* = 0.01), 40 minutes after awakening (*t*(17) = 2.4, *P* = 0.03), 60 minutes after awakening (*t*(17) = 2.4, *P* = 0.03), and 80 minutes after awakening (*t*(17) = 2.5, *P* = 0.02). None of the comparisons between cortisol levels for the Dim Light Session and Light Session 1 were statistically significant (*t*(17) = 2.1, *P* = 0.06 at 20 minutes after awakening, *t*(17) = 1.6, *P* = 0.1 at 40 minutes after awakening, *t*(17) = 1.3, *P* = 0.2 at 60 minutes after awakening, and *t*(17) = 0.5, *P* = 0.6 at 80 minutes after awakening).

## 4. Discussion

The impact of morning retinal light exposures on CAR in subjects who did not undergo sleep restriction has been demonstrated previously [22, 23]. The present results extend those previously published by demonstrating that short-wavelength light exposures upon awakening increase CAR and AUC in sleep-restricted adolescents.

Our results are not consistent with those from Jung et al., who showed a decrease in morning cortisol levels after light exposure [24]. The present study investigated the impact of 470-nm light on CAR, while the study by Jung et al. investigated the impact of a polychromatic (white) light on the ascending and descending segments of the cortisol rhythm. Moreover, the very different experimental protocols employed in the two studies may contribute to these different results. Jung et al. kept participants in the laboratory for several days, while our participants came to the laboratory once a week for an overnight session. They exposed participants to 10,000 lux of a white light (4100 K fluorescent lamp) for 6.5 hours, while we exposed our participants to 40 lux of a 470-nm light for 80 minutes. Our participants, although sleep restricted, received the light exposure upon awaking from a 4.5 hr sleep opportunity, while participants in the Jung et al. experiment had been awake for a few hours prior to the time when CAR would have occurred if the subjects had been asleep [24]. It is known that CAR is highly correlated with waking, and it may be that the timing of sleep and waking modulates the impact of light on CAR.

It was expected that cortisol levels in Light Sessions 1 and 2 would be similar because the experimental conditions presented to the participants were the same in the mornings. Interestingly, the higher cortisol levels in Light Session 2 than in Light Session 1 may have been due to the differences in the previous night light exposures, namely, subjects were exposed to long-wavelength light and to dim light the evening before Light Sessions 1 and 2, respectively. It is also not known why the variance in Light Session 2 was greater than in Light Session 1. As mentioned above, long-wavelength light in the evening was used because it was not expected to phase shift the circadian clock, but it was hypothesized to increase performance because of its alerting effects. It is known that the response of the pineal gland will be modulated by prior light exposure [30, 31] but nothing is known about the adaptation of the adrenal gland to prior light exposure. Future studies should be performed to directly investigate if evening long-wavelength light (which is not expected to impact circadian phase) can modulate CAR to light.

From the present results, it is not possible to determine whether the melatonin pathway was involved in the enhanced CAR response to short-wavelength light. Evidence that light in the middle of the night can impact cortisol levels via pathways other than melatonin suppression has been demonstrated [32]. It would be interesting to investigate if long-wavelength light can also enhance CAR or if the mechanisms by which light impacts the adrenal gland are different at different times of the night (i.e., middle of the night versus morning). In other words, the SCN may simply serve as a gating mechanism for the adrenal response to light at different times of the day [33]. In fact, studies have linked changes (both increases and decreases) in adrenal sensitivity to adrenocorticotropic hormone with time of day and the SCN [34–36]. Consistent with the idea that the sensitivity of the adrenal gland to light may change over the course of the day, Leproult et al. showed that morning light increased cortisol levels, but evening light did not [22].

Previous studies have suggested that there is a relationship between CAR and chronotypes. Morning chronotypes were shown to have higher CAR than evening chronotypes [37]. Our *post hoc* analyses did not show any relationship between CAR and chronotypes, nor did it show any age and gender differences in light responses. This lack of relationship between chronotypes and CAR may be because the majority of our subjects reported being a 3 or 4 on the MCTQ (*n* = 13), while 4 subjects reported being a 1 and one subject reported being a 5. Further studies with a larger sample size should be performed to specifically investigate how chronotypes, age, and gender may differentially impact the effect of short-wavelength light on CAR.

## 5. Conclusions

The present results are the first to show that 40 lux of short-wavelength (blue) light enhances CAR in adolescents who were restricted from sleep for one night (with 4.5 hours allowed in bed). An enhancement of CAR in humans by morning light exposure, especially in adolescents who tend to be sleep-deprived, may be important to “stimulate” the body when it is time for it to be active and, thus, prepare adolescents for any environmental stress they might experience [38]. In addition, data presented here tentatively suggest that reduced evening light exposures may also influence CAR. Further studies should be performed to confirm these findings.

## Figures and Tables

**Table 1:** Chronotypes of each participant.

Subject	Age (years)	Chronotype
	Females	
1	13	3
2	12	3
3	12	3
4	13	4
5	15	3
6	14	3
7	14	4
8	14	3
9	14	3
Female mean ± SD	13.4 ± 1.0	3.2 ± 0.4

	Males	
10	17	1
11	17	1
12	17	1
13	16	4
14	15	4
15	17	4
16	17	3
17	16	5
18	17	1
Male mean ± SD	16.5 ± 0.7	2.7 ± 1.7

mean average ± SD	15 ± 1.8	2.9 ± 1.2

**Table 2:** Lighting scenarios presented to all 18 participants in a counterbalanced manner.

Lighting scenarios		
	Evening	Morning
Dim Light Session	<5 lux at the cornea from an incandescent light source	<5 lux at the cornea from an incandescent light source
Light Session 1	40 lux(0.182 W/m^2^)of long-wavelength, red light (*λ*_max_≈630 nm)	40 lux(0.401 W/m^2^)of short-wavelength, blue light (*λ*_max_≈470 nm)
Light Session 2	<5 lux at the cornea from an incandescent light source	40 lux(0.401 W/m^2^)of short-wavelength, blue light (*λ*_max_≈470 nm)
